# In Vivo Study of Intra-articular Tualang Honey Formulation as a Potential Alternative to Synvisc® in an Osteoarthritic Rabbit Model

**DOI:** 10.5704/MOJ.2603.017

**Published:** 2026-03

**Authors:** AR Nour-El-Huda, Z Ahmad-Hafiz, NH Mohd-Jan, NF Mohamad-Amri, MZ Ibrahim, M Farahidah, SA Abu-Hassan, MH Nasuruddin

**Affiliations:** 1 Department of Basic Medical Sciences, International Islamic University Malaysia, Kuantan, Malaysia; 2 Department of Orthopaedics, Traumatology and Rehabilitation, International Islamic University Malaysia,Kuantan, Malaysia; 3 Department of Pharmaceutical Technology, International Islamic University Malaysia, Kuantan, Malaysia

**Keywords:** Tualang honey, synvisc, osteoarthritis, rabbit model, viscosupplement

## Abstract

**Introduction:**

Osteoarthritis (OA) is a degenerative joint disorder characterised by cartilage degradation and inflammation. This study evaluates the efficacy of Tualang Honey Formulation (THF) (Patent no. MY-179303-A), a natural-based formulation tested for biocompatibility and toxicity, compared to Synvisc, a conventional hyaluronic acid injection, in managing OA in vivo using an animal model.

**Materials and methods:**

Twenty-eight New Zealand White Rabbits (NZWR) were operated to surgically induce OA and divided into three groups: sham group (n=1), THF-treated group (n=3) and Synvisc-treated group (n=3). Treatments were administered intra-articularly three weeks post-surgery. Radiographic assessments were performed at 3, 6, 12, and 24 weeks. Histological analyses at 24 weeks utilised Masson-Goldner trichrome staining.

**Results:**

In the sham group, severe cartilage loss, thinning, and thickened subchondral bone were observed, reflecting progressive OA. Radiographically, both THF and Synvisc groups demonstrated improved joint space and reduced subchondral sclerosis. At six weeks, the THF and Synvisc groups exhibited improvements from three weeks. At 12 weeks, the THF group demonstrated better joint space preservation, while outcomes were comparable at 24 weeks. Histologically, the THF group displayed thicker articular surfaces with signs of bone remodelling with better cartilage preservation. In contrast, Synvisc-treated joints displayed thinner, irregular cartilage and subchondral bone changes, suggestive of potential bone cyst formation.

**Conclusion:**

THF is equally effective as Synvisc in reducing OA progression while showing additional benefits in cartilage preservation and bone remodelling. As a natural and local product, THF represents a promising alternative viscosupplement for OA management, warranting further clinical validation.

## Introduction

Osteoarthritis (OA) is one of the most prevalent degenerative joint diseases, leading to chronic pain, joint dysfunction, and significant disability^1,2^. The progressive nature of OA, characterised by cartilage degradation, subchondral bone remodelling, osteophyte formation, and synovial inflammation, poses a substantial burden on individuals and healthcare systems worldwide^3,4^. In 2019, the World Health Organization reported that OA affects an estimated 520 million people worldwide, particularly in weight-bearing joints such as the knee and hips. While OA is considered a degenerative disease, its incidence sharply increases with age^5^. The pathophysiology of OA is complex and multifactorial. Mechanical stress, obesity, ageing, and genetic predisposition are among the most well-recognised contributors^6^. At the cellular level, OA involves a cascade of biochemical and biomechanical events that disrupts articular cartilage homeostasis^7^. Increased oxidative stress and the release of proinflammatory cytokines, such as interleukin-1β (IL-1β) and tumour necrosis factor-alpha (TNF-α), exacerbate cartilage breakdown^8^.

Management of OA primarily focuses on symptom relief and the preservation of joint function, as there is no definitive cure. Nonsteroidal anti-inflammatory drugs (NSAIDs) remain the cornerstone of pharmacologic therapy, providing pain relief and reducing inflammation. However, long-term NSAID use is associated with adverse effects, including gastrointestinal ulcers, cardiovascular complications, and renal toxicity^9^. Intra-articular corticosteroids are also used for their anti-inflammatory properties. However, their repeated use can accelerate cartilage damage and is not superior to other interventions, such as intra-articular hyaluronic acid injections or physiotherapy in the long term^10^. Viscosupplementation with hyaluronic acid-based products has been used for over 50 years as an option in OA management^11^. These injections aim to restore the viscoelastic properties of synovial fluid, improving joint lubrication and shock absorption^12^. Synvisc One® (Hylan G-F 20) [Genzyme, Netherlands] has effectively reduced pain severity, stiffness, and difficulties in daily activities associated with knee osteoarthritis^13^. Despite its proven efficacy and cost-effectiveness in previous research^14^, the high cost and synthetic composition have driven interest in exploring natural and cost-effective alternatives.

Scientifically, honey has been extensively studied for its medicinal properties, including antioxidative, anti-inflammatory, and antimicrobial activities. These characteristics, resulting from the rich composition of phenolic acids, flavonoids, and vitamins, make it a promising candidate for managing chronic inflammatory conditions like OA. Tualang Honey (TH), sourced from the giant Asian honeybees *(Apis dorsata)*, which build their hives in the Tualang trees *(Koompassia excelsa)*, one of the tallest trees in the Southeast Asian rainforests, has garnered attention for its exceptional therapeutic potential. Studies have demonstrated that TH has higher levels of antioxidants than other types of honey, primarily due to its unique floral sources and environmental conditions. These antioxidants neutralise reactive oxygen species (ROS) and reduce oxidative stress, which are known to be involved in cartilage degradation in OA. A review by Martínez-Armenta *et al*^15^ identified bioactive compounds in honey, including flavonoids such as chrysin and quercetin, as well as phenolic acids like gallic acid and caffeic acid. These compounds have been shown to regulate articular homeostasis, inhibit inflammation, and counteract oxidative stress, thereby potentially slowing the progression of osteoarthritis. Their findings suggest that honey formulations may serve as a complementary option to conventional OA treatments.

The anti-inflammatory properties of honey help modulate cytokine production, reducing the inflammatory cascade in the joint microenvironment. Importantly, previous studies have demonstrated that honey could alleviate symptoms associated with OA changes in animal models, indicating its potential to provide relief for those suffering from the disease^16,17^. The preclinical findings demonstrated that oral administration of Tualang honey significantly reduces joint inflammation, pain hypersensitivity, and cartilage degradation, highlighting its potential as a disease-modifying agent. These promising outcomes provide a strong rationale for continuing to investigate honey-based formulations as alternative or adjunct therapies for OA.

The growing prevalence of OA and the limitations of existing therapies indicate a need for innovative approaches that combine efficacy, safety, and accessibility. Tualang Honey has been identified for its potential therapeutic properties, which may bridge this gap^18^. However, it is necessary to conduct rigorous preclinical and clinical evaluations to determine its efficacy and safety as a therapeutic option for OA. This study examines the effectiveness of Tualang Honey Formulation (Patent no. MY-179303-A) in comparison to Synvisc hyaluronic acid in a controlled rabbit model of OA. Although Synvisc (Hylan G-F 20) is not classified as a disease-modifying osteoarthritis drug (DMOAD), it has been widely used in clinical settings to provide symptomatic relief, particularly for pain and stiffness as a viscosupplement. It was chosen as a comparative reference to evaluate whether the THF could provide similar or improved structural outcomes.

This study aimed to evaluate radiological and histological changes as indicators of disease progression and to provide evidence of the therapeutic potential of the intervention.

## Materials and Methods

This experimental study used 24 New Zealand White Rabbits (NZWR) weighing 2.5–3.5 kg. It was conducted at the Orthopaedic Research Laboratory (ISO/IEC 17025:2017), Department of Orthopaedic, Traumatology and Rehabilitation, Kulliyyah of Medicine, International Islamic University Malaysia (IIUM), Kuantan, Pahang. The ethical approval was obtained from the IIUM Research Animal Care and Use Committee (IACUC 2016-010). All experiments were conducted under the IIUM Integrated Centre for Research Animal, Care and Use (ICRACU) guidelines.

Tualang Honey Formulation (THF) is a specialised formulation of Tualang Honey developed and patented under the Intellectual Property Corporation of Malaysia (MY-179303-A). The prepared THF was pre-filled into sterile syringes and subjected to gamma radiation for sterilisation. Biocompatibility and toxicity testing conducted at Makmal Bioserasi, Universiti Kebangsaan Malaysia, confirmed that the THF was safe and non-toxic. These assessments ensured the safety and suitability of THF for intra-articular administration.

The rabbits were weighed and anaesthetised with an intravenous injection of ketamine hydrochloride (KTX) [Pfizer, United Kingdom] administered with an initial dose of 0.2ml/kg and subsequently maintained at 1.0ml/kg. The rabbit OA knee induction in our study utilised the method described by Arzi *et al*^19^. The skin at the incision site was shaved and cleaned with povidone-iodine, followed by draping with a sterile surgical cloth. A medial parapatellar longitudinal incision was executed to provide access to the knee joint, where retractors were used to expose the medial and lateral condyles. Medial and lateral meniscectomies were performed, and the condyles were scraped five times each to induce OA. Haemostasis was secured, and the surgical wound was closed in layers in a simple continuous suture with 4-0 Monosyn sutures. The surgical site was dressed with povidone-iodine and covered with gauze. The knee joints were bandaged to protect them from access by the animal. Post-operatively, the rabbits were monitored within their cages for bleeding. The post-operative care included daily administration of intramuscular injection of enrofloxacin [0.1mL/kg; Batril® 5%, Bayer AG, Leverkusen, Germany] as an antibiotic prophylaxis and tramadol hydrochloride [0.02ml/kg; Mabron, Medochemie LTD, Limassol-Cyprus] for analgesia. Tramadol was administered for seven consecutive days to prevent wound infection and manage post-operative pain. Throughout the 24-week study period, all animals were monitored daily to assess general health, mobility, wound condition, and signs of discomfort.

**Fig. 1:**
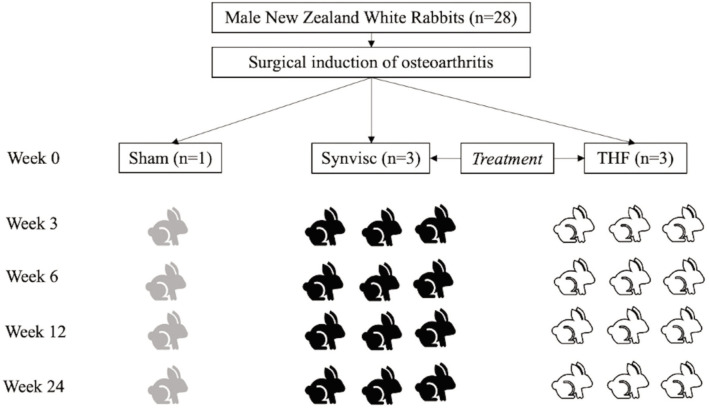
Study design outlining the experimental groups and timeline. Male New Zealand White Rabbits (n=28) were surgically induced with osteoarthritis (OA) and divided into three groups: sham (n=1), THF-treated (n=3), and Synvisc-treated (n=3). Intra-articular injections of 0.2mL THF or Synvisc were administered three weeks post-surgery in the treatment groups. Radiographic assessments were conducted at 3, 6, 12, and 24 weeks after treatments, while histological evaluations were performed at the same intervals. Rabbits were sacrificed in subgroups at each evaluation point to assess disease progression and treatment effects.

The study included three experimental groups: sham, THF-treated, and Synvisc-treated, each subdivided into four evaluation subgroups based on intervals at 3, 6, 12, and 24 weeks (Fig. 1). Male NZWR underwent OA induction surgery, after which intra-articular injections were administered three weeks post-surgery. The THF-treated group received 0.2mL of Tualang Honey Formulation, while the Synvisc-treated group received 0.2mL of Synvisc-One® (Hylan G-F 20) [Genzyme, Netherlands]. The sham group served as the control and did not receive any therapeutic intervention.

Radiological assessments were conducted to evaluate OA progression. Anteroposterior and lateral radiograph imaging of the knee joints was performed at specified intervals (weeks 3, 6, 12, and 24) to assess changes in joint structure. The radiographs were made under consistent conditions of the tube to plate distance at 70cm, 63kV, 2.00mA, and 2.00ms. The computed radiography systems used [Philips Optimus 80, Philips Medical System, USA] were equipped with a high-speed reader [Regius 190, Konica Minolta] and laser printer [Drypro 793, Konica Minolta]. The radiological parameters included the presence of joint space narrowing, subchondral sclerosis, and the formation of osteophytes. The acquired images were analysed to monitor disease progression and to compare the outcomes between the sham, THF-treated, and Synvisc-treated groups. The evaluation of the radiograph films was performed independently by two orthopaedic surgeons blinded to the treatment received by the rabbits.

Upon completion of the radiological assessment, three animals were sacrificed at specified intervals (weeks 3, 6, 12, and 24), and the injected knees were excised en bloc. Tissue specimens were fixed, decalcified, and processed for histological evaluation. The samples were examined by using a transmitted light research motorised microscope [Nikon Eclipse Ni, Japan] and analysed using NIS-Elements Software analyser. The Masson-Goldner trichrome staining technique assessed cartilage structure, subchondral bone changes, and matrix integrity. A blinded anatomist reviewed the specimens and compared changes between the sham, THF-treated, and Synvisc-treated groups. Similarly, the reviewer was also unaware of the type of treatment received by the animals.

## Results

**Fig. 2:**
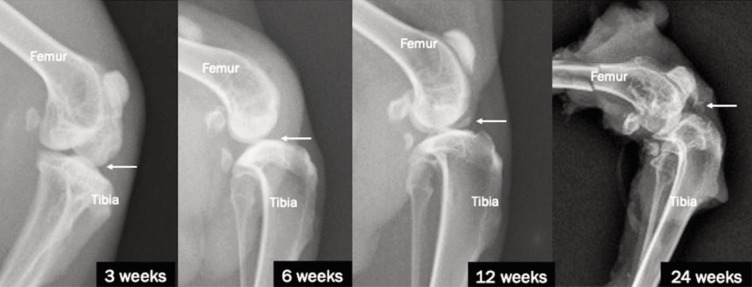
Lateral view radiograph of the knee joints of NZWR at 3, 6, 12, and 24 weeks for sham groups.

**Fig. 3:**
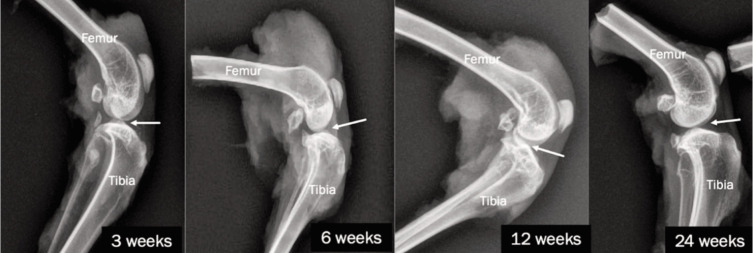
Lateral view radiograph of the knee joints of NZWR at 3, 6, 12, and 24 weeks for THF-treated groups.

**Fig. 4:**
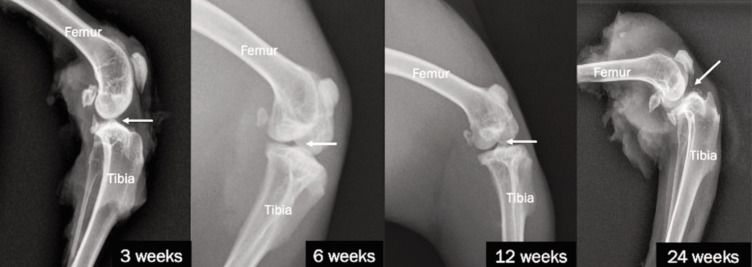
Lateral view radiograph of the knee joints of NZWR at 3, 6, 12, and 24 weeks for Synvisc groups.

**Fig. 5:**
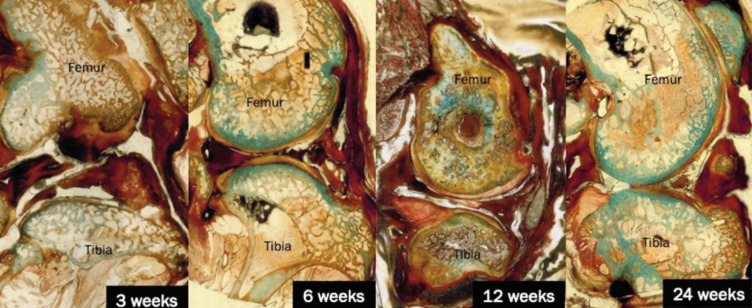
Histological images showing cartilage and bone structure at 3, 6, 12, and 24 weeks for sham groups. Masson-Goldner Trichrome staining. Original magnification X40.

**Fig. 6:**
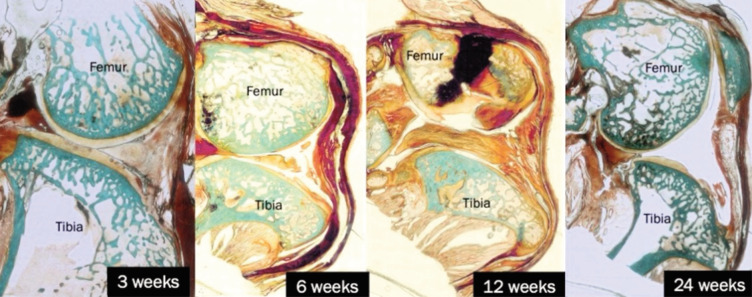
Histological images showing cartilage and bone structure at 3, 6, 12, and 24 weeks for THF-treated groups. Masson-Goldner Trichrome staining. Original magnification X40.

**Fig. 7:**
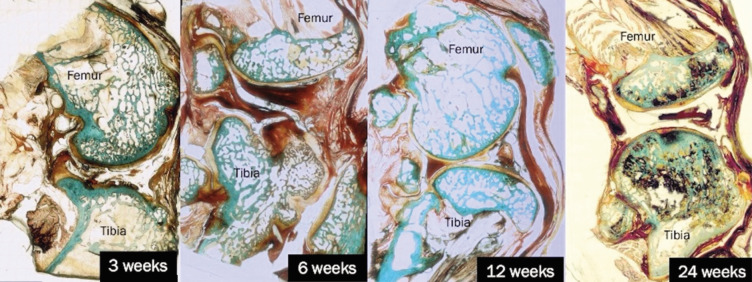
Histological images showing cartilage and bone structure at 3, 6, 12, and 24 weeks for Synvisc groups. Masson-Goldner Trichrome staining. Original magnification X40.

The progression of osteoarthritis across the sham, THF, and Synvisc groups was analysed radiographically as shown in Fig. 2 to 4. Complementary histological findings are presented in Fig. 5 to 7 to evaluate the differences in joint changes over time across 3, 6, 12, and 24 weeks.

In the sham group (Fig. 2), radiographs demonstrated progressive osteoarthritic changes without therapeutic intervention. At three weeks, the joint surfaces appeared relatively smooth with no significant osteophyte formation or erosion, though mild joint space narrowing was observed. By six weeks, there was a noticeable reduction in joint space accompanied by the development of early osteophytes and mild irregularities in the articular surfaces. At 12 weeks, the narrowing of the joint space became more pronounced, with visible subchondral sclerosis and osteophyte formation along the joint margins. Evidence of surface irregularities and increased density in subchondral bone was also noted. By 24 weeks, the progression was marked by severe narrowing of the joint space, extensive osteophyte formation, and advanced subchondral sclerosis. Subchondral cysts were present, reflecting significant degenerative changes.

Fig. 3 shows the radiographic findings in the THF-treated group. At three weeks, the joint demonstrated smooth and intact articular surfaces with no significant erosion or presence of osteophytes, and the joint space appeared normal. At six weeks, a marked reduction in joint space and irregularities in the articular surfaces were observed. At 12 weeks, while joint space remained reduced, there were no signs of osteophyte, sclerosis, or cystic changes. At 24 weeks, there was a further reduction in joint space, particularly on the medial side, with osteophyte formation present. There were no cysts or subchondral sclerosis observed.

The radiographic changes of the Synvisc-treated group are shown in Fig. 4. At three weeks, the joint space was well preserved, characterised by normal smooth articular surfaces and the absence of erosive changes or osteophyte formation. By six weeks, however, the knee joint showed decreased joint space accompanied by significant subchondral sclerosis and osteophyte development. At 12 weeks, the reduction of joint space was pronounced, with osteophyte formation and subchondral sclerosis noticeable. Finally, at 24 weeks, the joint exhibited severe narrowing with the presence of osteophytes, subchondral sclerosis, and subchondral cysts indicative of advanced degenerative changes.

Fig. 5 illustrates the histological progression in the sham group over time. At three weeks, the articular surface exhibited a relatively smooth appearance, with minimal surface irregularities. However, early signs of cartilage loss were noted, likely due to surgical intervention. By six weeks, there was evident articular cartilage thinning. At 12 weeks, the underlying bony tissues appear thickened. By the 24-week assessment, the joint structure showed severe cartilage loss, thinning of the cartilage and the presence of fissures, which indicated advanced osteoarthritic changes in the absence of therapeutic intervention.

In Fig. 6, the histological changes in the THF-treated group are illustrated across the same time intervals. At three weeks, the articular surface exhibited a relatively smooth appearance, although a few vertical fissures were noted, likely due to the surgical intervention. The cartilage matrix appeared denser and better preserved compared to the sham group. By the six-week evaluation, there was a marked degeneration of articular cartilage on both articulating bones, characterised by minimal surface irregularities on the articular and some disintegration of cartilage. However, the cartilage appeared thicker and less irregular compared to the sham group. At 12 weeks, the articular surface displayed significant irregularities, with cartilage disintegration and persistent vertical fissures. By the 24-week assessment, the THF-treated group displayed a thickened articular surface characterised by irregularity that may indicate new bone growth, potentially in the form of osteophytes, along with a thin articular cartilage overlying these structures. The cartilage erosion appeared less severe compared to the sham group.

Fig. 7 presents the histological findings for the Synvisc-treated group across 3, 6, 12, and 24 weeks. At three weeks, the cartilage surfaces appeared disrupted and irregular due to disintegration with thickening of the underlying bone tissue. The disruption was more pronounced than in the THF-treated group, but slightly better than in the sham group. The six-week assessment indicates persistent irregularities in the cartilage, accompanied by thickening of the bony tissues, while the adjacent articular surface remained relatively intact. Compared to the sham group, cartilage degeneration appeared slightly reduced, but still irregular. By 12 weeks, the cartilage showed irregularities and thinning, consistent with ongoing disintegration. There were fewer cartilage cells and more visible damage compared to the THF-treated group. The assessment at 24 weeks revealed areas of thin articular cartilage, with changes in the adjacent subchondral bone suggesting the possibility of bone cyst formation within the joint cavity. The remaining thicker articular cartilage areas still contained chondrocytes within their lacunae. These thicker areas were more in the Synvisc-treated group compared to the sham group, but they were not well preserved as in the THF-treated group.

## Discussion

This study demonstrates that intra-articular injections of THF were comparable to Synvisc in facilitating the progression of OA in a rabbit model. Over 24 weeks, both interventions demonstrated significant improvements in the radiological assessment of OA, specifically in the joint space width and subchondral sclerosis. Notably, at 12 weeks of evaluation, the THF group exhibited a trend toward better joint space preservation. These findings align with a previous study reported by Jimoh-Abdulghaffaar *et al*^16^ which indicated that honey could improve radiographic outcomes in animal models of OA, reflecting its potential symptomatic relief in this condition.

Although OA progression in rabbits occurs over a shorter timescale compared to humans, several studies have validated the 24-week post-operative period as representative of advanced-stage OA in human knees. Structural degeneration observed at this time point, including marked cartilage erosion, fissuring, osteophyte formation, and subchondral bone remodelling. These changes closely mimic the pathological features of late-stage human OA^20^. A systematic review by Oláh *et al*^21^ compared animal models of post-traumatic knee osteoarthritis. The report showed rabbits exhibit consistent and progressive cartilage and subchondral bone changes within 12 to 24 weeks after meniscal injury. Additionally, biochemical markers of cartilage collagen degradation have been shown to increase during OA progression in rabbits over a 20-week period. This reflects cartilage breakdown patterns similar to those observed in human patients^22^. These findings support the relevance of the 24-week rabbit model. It reproduces the structural and biochemical changes of OA that correspond to a valid human translational model for studying chronic structural changes in OA and their therapeutic effects.

The sham group findings provided critical insights into the natural progression of OA in the absence of therapeutic interventions. Radiologically, the sham group exhibited progressive joint space narrowing, subchondral sclerosis, and osteophyte formation over the 24-week study period. These degenerative changes are consistent with advanced OA pathology observed in untreated animal models and human studies^23^. Joint space narrowing is a hallmark radiographic feature of OA, indicates cartilage degradation, while subchondral sclerosis and osteophyte formation indicate adaptive bone remodelling processes that contribute to disease progression^24^. Histologically, the sham group demonstrated loss of cartilage and thickened subchondral bone, which are characteristic features of OA progression^23^. The pronounced degenerative changes observed in the sham group highlight the progressive nature of OA in the absence of therapeutic intervention. These observations provide a critical baseline for evaluating the efficacy of therapeutic interventions such as THF and Synvisc.

Synvisc demonstrated a partial protective effect on the cartilage, with some sections displaying smooth and regular cartilage. Nonetheless, there were evident thinning and irregularities in the cartilage along with signs of subchondral bone changes, such as potential cyst formation^25^. These observations are consistent with the established mechanism of viscosupplementation of hyaluronic acid, which enhances joint lubrication and decreases frictional stress on the cartilage^26^. Despite these benefits, Synvisc, as an intra-articular injection of hyaluronic acid, does not fully prevent cartilage degeneration as highlighted by previous studies^27^.

In contrast, the THF-treated group displayed distinct histological features suggestive of cartilage preservation and bone remodelling. In the earlier stages of the study, THF-treated joints exhibited relatively smooth cartilage surfaces with minimal irregularities. However, by 24 weeks of evaluation, there were signs of thickened articular surfaces and potential osteophyte formation, which could reflect reparative bone remodelling and mechanical adaptation^28^. Despite some cartilage irregularities and thinning, the overall preservation of cartilage was superior in the THF-treated group compared to Synvisc. This observation aligns with the bioactive properties of honey, particularly its antioxidative and anti-inflammatory effects, which may play a crucial role in decreasing cartilage degradation and stimulating reparative mechanisms within the joint^29^. Additionally, the antioxidative and anti-inflammatory properties of THF, attributed to flavonoids and phenolic acids, likely play a pivotal role in mitigating cartilage degradation by reducing oxidative stress and inhibiting pro-inflammatory cytokines^15^.

Overall, both therapeutic approaches exhibited varying degrees of cartilage preservation, revealing distinct effects of their potential roles. However, THF demonstrated a trend toward superior cartilage integrity and enhanced adaptive bone remodelling, while Synvisc appeared more effective in reducing subchondral bone alterations. These differences suggest that the two therapeutic options may operate through complementary mechanisms, highlighting the need for further research to optimise their application, whether used independently or in combination, to address the multifaceted pathophysiology of osteoarthritis^30^.

Additionally, THF presents several advantages over Synvisc as a viscosupplement for the management of OA. Its natural composition makes it an appealing choice for patients seeking a halal and organic viscosupplement option, especially in regions where cultural and religious values heavily influence healthcare decisions^31^. Moreover, THF is likely to be a more accessible option for managing OA in low- and middle-income settings compared to synthetic hyaluronic acid-based viscosupplements^14^. The absence of adverse effects in this study suggests the safety profile of THF, presenting it as a natural and biocompatible alternative with minimal complications^32^.

Although the THF used in this study has not yet been tested in human subjects, evidence from a recent comprehensive systematic review of Tualang honey highlights its broad therapeutic potential^18^. This review compiled findings from 123 studies, including in vitro, in vivo, and randomised controlled trial research, demonstrating that Tualang honey exhibits a wide range of pharmacological activities, including antimicrobial, anticancer, anti-inflammatory, antioxidant, antinociceptive, and neuroprotective effects. While THF itself has not yet undergone clinical evaluation, preclinical studies in osteoarthritic animal models support the oral efficacy of honey in reducing joint inflammation, pain, and cartilage degradation^16,17^. While no clinical trials have yet investigated THF specifically, a recent human study utilising a honey-ginger oral syrup demonstrated symptom improvement in knee osteoarthritis, suggesting translational relevance for honey-derived therapies^33^. These findings reinforce the potential of THF as a safe and effective natural adjunct for osteoarthritis management, warranting future clinical investigation.

Despite its potential, this study has several limitations that should be addressed in future research. Firstly, the findings are derived from a rabbit model of OA, which, although valuable, may only partially replicate the complex pathophysiology of human OA^34^. Secondly, the study predominantly focused on radiographic outcomes, with limited exploration of biochemical and histopathological markers of cartilage health. Incorporating these additional analyses would provide a more comprehensive understanding of the therapeutic efficacy of THF. Finally, the long-term systemic effects of repeated intra-articular THF injections remain unresolved, highlighting the necessity for further investigations to assess their safety and efficacy over prolonged use.

Future research should focus on clinical trials aimed at validating the effectiveness and safety profile of THF in human OA patients. Advanced imaging modalities, such as MRI, could provide a more detailed understanding of cartilage quality and subchondral bone health, complementing radiographic assessments. Additionally, investigating inflammation and cartilage metabolism biomarkers could help reveal the molecular mechanisms underlying the therapeutic effects of THF. Lastly, optimising the formulation of THF, such as adjusting its dosing frequency or synergistically combining it with other bioactive agents, could enhance cartilage protection while minimising adverse effects on bone health. These efforts will help in establishing THF as a sustainable and effective therapeutic option for osteoarthritis in clinical settings.

## Conclusion

This study demonstrated that THF is as effective as Synvisc in mitigating osteoarthritis (OA) progression, with both therapeutic approaches showing significant improvements in radiographic indicators such as joint space width and subchondral sclerosis over 24 weeks. The natural composition of THF, affordability, and safety profile highlight its potential as an accessible and sustainable alternative to synthetic viscosupplements, particularly in regions where cultural and financial barriers limit the use of conventional therapies. With no significant adverse effects reported, THF represents a promising therapeutic option for OA patients who may seek natural, biocompatible, and halal-certified therapeutic options. This is especially relevant for populations in Muslim-majority countries, where halal-certified medical products are increasingly in demand and could address both therapeutic and ethical considerations. THF could provide a cost-effective solution for OA management in resource-limited healthcare settings. Unlike synthetic hyaluronic acid-based products such as Synvisc, often associated with high costs and limited accessibility, THF offers an affordable alternative that may enhance access to effective therapeutic solutions of OA for underprivileged populations. Further research, including large-scale human clinical trials and long-term safety evaluations, is essential to validate the findings and pave the way for the adoption of THF in global markets. By bridging the gap between modern medical advances and cultural sensitivities, THF could transform OA management and contribute to the worldwide movement toward inclusive and sustainable healthcare solutions.
